# Is There a Carcinogenic Risk Attached to Vitamin B_12_ Deficient Diets and What Should We Do About It? Reviewing the Facts

**DOI:** 10.1002/mnfr.202000945

**Published:** 2021-02-19

**Authors:** Alexandra K. Loedin, Dave Speijer

**Affiliations:** ^1^ Amsterdam UMC, Medical Biochemistry University of Amsterdam The Netherlands

**Keywords:** Cancer, DNA methylation, DNA synthesis, veganism, vitamin B_12_ deficiency

## Abstract

The number of individuals partaking in veganism has increased sharply in the last decade. Therefore, it is critical to look at the implications of vegan diets for public health. Although there are multiple health benefits of a vegan diet, studies have also linked the diet with deficiencies in various micronutrients. This study focuses on vitamin B_12,_ because of its critical role in DNA synthesis and methylation. In light of these connections, a critical review of recent scientific literature is conducted to understand the effects of a B_12_ deficient diet on the genome and epigenome, and whether it can give rise to cancer. It is observed that a B_12_ deficiency leads to increased uracil misincorporation, leading to impaired DNA synthesis and genomic instability. The deficiency also leads to global hypomethylation of DNA, a hallmark of early carcinogenesis. The findings of this study highlight the need for increased awareness among vegans to ensure adequate B_12_ intake through supplementation or consumption of fortified products as a preventative measure. Additionally, the biofortification of staple crops and an improved version of fermented products with increased B_12_ content can be developed when inadequate intake seems otherwise inevitable.

## Graphical abstract

Percentages of those partaking in veganism are rising. Vegan diets can result in insufficient levels of vitamins, such as B_12_, critical for DNA synthesis and methylation. Scientific literature shows B_12_ deficient diets to influence the (epi)genome and thus increase cancer risk. We conclude that vegans should make sure of sufficient B_12_ intake.

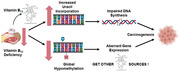

## 1 Introduction

Although the concept of a plant‐based diet is not novel, recently an impressive rise in individuals adopting a vegan diet has been observed globally. Veganism is defined as the practice of not consuming animal‐derived products, such as meat, fish, poultry, eggs, dairy products, and honey.^[^ ^1^ ^]^ Followers of the vegan diet are commonly referred to as “vegans” and this terminology will be used in this review. Taking the United States as an example, a recent study showed that 6% of the total population identified as followers of the vegan diet,^[^ ^2^ ^]^ of which 11% are young adults (age 25–34).^[^ ^3^ ^]^ Similarly, there was an increase of 45,0000 vegans in the United Kingdom in the last 5 years.^[^ ^4^ ^]^ In Portugal, there were 12,0000 individuals who claimed to follow a plant‐based diet in 2018, compared to a mere 30,000 in 2007.^[^ ^2^ ^]^ These statistics could be attributed to studies linking a vegan diet with improved health, sustainability, and reducing the global carbon footprint.^[^ ^3^, ^5^, ^6^, ^7^, ^8^ ^]^ Despite its benefits, plant‐based diets tend to result in a deficiency of certain micronutrients, namely iron, zinc, calcium, vitamin D, omega‐3 (*n‐*3) fatty acids, iodine, and vitamin B_12_ (B_12_).^[^ ^3^ ^]^

This review will focus on B_12_, also known as cobalamin, an essential micronutrient that is present in meat, fish, egg, and dairy products.^[^ ^9^, ^10^ ^]^ B_12_ is produced by microorganisms that are present in the bowel of ruminants (e.g., cattle, sheep, etc.) and omnivorous animals (e.g., chicken, pigs). In aquatic environments such microorganisms are found in phytoplankton, which are consumed by fishes and crustaceans. Once absorbed in the small intestine, the vitamin is then stored in the liver and muscle of these animals or secreted into milk.^[^ ^11^ ^]^ This water‐soluble vitamin acts as a co‐factor for methionine synthase (MS; the enzyme that catalyzes the conversion of homocysteine to methionine) and L‐methylmalonyl‐CoA mutase (the enzyme that catalyzes the conversion of methylmalonic acid (MMA) to succinyl‐CoA). Thus, B_12_ plays a crucial role in red blood cell production, neurologic development and function, DNA synthesis, and DNA methylation.^[^ ^12^ ^]^ A B_12_ deficiency is correlated with both aberrant DNA synthesis and methylation.^[^ ^13^ ^]^ Errors in these physiological processes lead to a variety of clinical manifestations, resulting in megaloblastic anemia, neurological impairment, cognitive decline, and neurodevelopmental disorders.^[^ ^14^, ^15^ ^]^ Therefore, it is imperative to have sufficient amounts of B_12_ in one's diet.

Multiple studies have shown that due to the dietary intake restrictions of a vegan diet, vegans have the highest risk of developing a B_12_ deficiency, in comparison to those consuming other vegetarian diets or omnivorous diets. In these studies, a B_12_ serum deficiency is defined as having less than 200 pmol L^‐1^ of B_12_.^[^ ^16^, ^17^ ^]^ In the United States, the prevalence of a B_12_ deficiency in young adults (age 20–39) was less than 3%; however, amongst vegans, the prevalence was a stunning 43%.^[^ ^16^, ^18^ ^]^ Furthermore, individuals who adopted a vegan diet at a younger age were found to have a higher risk of depleted B_12_ levels later in life.^[^ ^16^ ^]^ Having a deficiency at a young age could take a few years to trigger the onset of clinical manifestations.^[^ ^19^ ^]^ Previous research focused heavily on the effects of maternal B_12_ status on neonatal development.^[^ ^20^, ^21^, ^22^ ^]^ However, concurrent with the rising percentage of vegans among the younger demographic, research focus has shifted to how a B_12_ deficiency affects adult health.^[^ ^15^ ^]^

Recent studies have indicated a possible link between a B_12_ deficiency and the onset of cancer.^[^ ^23^, ^24^ ^]^ In 2019, cancer was considered one of the leading causes of death in high‐income countries, with almost twice as many deaths as from cardiovascular diseases, the leading cause of death worldwide.^[^ ^25^ ^]^ Despite the increasing amount of research, there is a substantial lack of systematic overviews regarding cancer as a potential clinical manifestation of a B_12_ deficiency. Therefore, this review aims to evaluate the effects of a B_12_ deficiency on both genome stability and the epigenome, providing a comprehensive overview of possible contributions to cancer development. We limited our sources to peer‐reviewed scientific papers, with the exception of online news articles from reputable websites for vegans (e.g., The Vegan Society).

The number of vegans, especially of the younger demographic, is predicted to further increase in the coming years due to the heightened awareness of the impact of non‐vegetarian, meat‐based diets on greenhouse gasses emissions, leading to climate change.^[^ ^3^ ^]^ Hence, it is critical to build an in‐depth understanding of the metabolic implications of this global dietary trend on individual health. We should also recognize the need for appropriate prevention strategies to ensure adequate B_12_ intake, mitigating the potentially adverse effects on public health.

## 2 Vitamin B_12_

**Figure 1:**
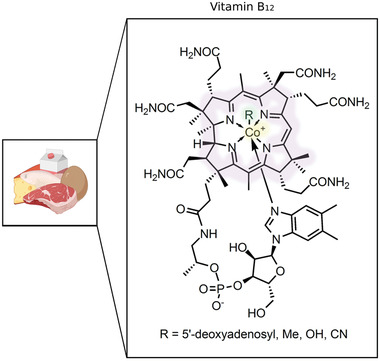
Chemical structure of B_12_. The structure of the vitamin is centered around a corrin ring (highlighted in purple) coordinately linked to a center cobalt atom (highlighted in yellow). There are four forms of B_12_, each differing in the functional group (highlighted in green) attached to the center cobalt atom. The R group could either be an adenosine (adenosylcobalamin), a methyl (methylcobalamin), a hydroxyl (hydroxocobalamin), or a cyanide group (cyanocobalamin), as indicated. Based on Jägerstad and Arkbåge, 2003.^[^ ^27^ ^]^

Though the vegan diet has benefits, it also has been linked to a deficiency of multiple essential micronutrients, B_12_ (see **Figure** **1**) amongst them.^[^ ^3^ ^]^ As described above, B_12_ is a water‐soluble vitamin found predominantly in animal derived dietary products. ^[^ ^14^ ^]^ B_12_ was first isolated in 1948 and later synthesized in 1973.^[^ ^26^ ^]^ It is part of the corrinoid family, as its chemical structure includes a corrin ring. This ring is made up of four reduced pyrrole rings, which are linked together in a macrocyclic ring, coordinately linked to a central cobalt atom. The structure of the corrin ring appears similar to the structure of heme in hemoglobin, apart from one less methylene bridge and the cobalt atom at its center. Also linked to the cobalt atom is a 5,6,‐dimethylbenzimidazole nucleotide part of the molecule.^[^ ^27^ ^]^ Four main active forms of B_12_ exist, cyanocobalamin, hydroxocobalamin, methylcobalamin (CH_3_•B_12_), and adenosylcobalamin; these differ in the “R” groups attached to the cobalt atom depicted in Figure 1. The three latter forms are typically found in animal products, while cyanocobalamin is the commercially available form used in supplements to prevent and treat a B_12_ deficiency.^[^ ^28^ ^]^

### 2.1 B_12_ Absorption

**Figure 2:**
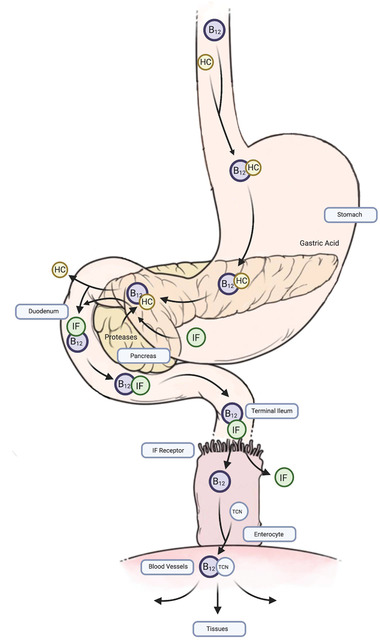
Absorption of B_12._ B_12_ gets extracted from food in the stomach by gastric juices and pepsin. It then binds to HC. Once the B_12_‐HC complex reaches the duodenum, HC is degraded by pancreatic proteases, and B_12_ binds to IF. In the terminal ileum, the B_12_‐IF complex is absorbed by the IF receptors, causing B_12_ to be released from IF. In the enterocyte, B_12_ then complexes with TC and is released into the circulation. This complex allows B_12_ to be absorbed by the tissues, especially the liver. Based on Lieberman and Marks, 2017.^[^ ^30^ ^]^

In the human body, B_12_ is extracted and absorbed from food in a series of steps that involve a complex network of proteins. Upon dietary intake, gastric acid and pepsin in the stomach release B_12_ from the proteins it was previously bound to. The vitamin then proceeds to attach to haptocorrin (HC), a glycoprotein primarily secreted by the salivary gland, until it reaches the duodenum. In the duodenum, HC is digested by pancreatic proteases, in the process releasing B_12_. Once released, B_12_ binds to intrinsic factor (IF), a glycoprotein secreted by gastric parietal cells. Binding to IF is essential for the absorption of B_12_ to occur, and an absence of IF leads to only 1–2% absorption of B_12_. Next, the vitamin is absorbed in the terminal ileum by IF receptors. ^[^ ^26^, ^28^, ^29^ ^]^ Once absorbed, B_12_ complexes with transcobalamin (TC) in the enterocyte, before release into the circulation (**Figure** **2**). This complex allows B_12_ to be delivered to the tissues; 50% of B_12_ is taken up by the liver.^[^ ^30^ ^]^ The body stores about 3–4 mg of B_12_ in the liver. This is a substantial amount and, if all dietary B_12_ uptake is discontinued, is sufficient for approximately 3 to 6 years before symptoms begin manifesting clinically. Thus, a B_12_ deficiency is relatively slow to develop.^[^ ^31^ ^]^ However, upon bariatric surgery, symptoms are observed earlier (most likely by a combination of interrupting the enterohepatic cycle and presurgical reductions in food intake).

### 2.2 The Role of B_12_

B_12_ is a fundamental nutrient, especially for two significant processes in the body, namely DNA synthesis and DNA methylation. Both processes form part of the one‐carbon metabolism pathway, a set of linked cyclical cytosolic reactions that result in the formation of methionine from homocysteine and donation of methyl groups. Methionine is crucial for the regeneration of S‐adenosylmethionine (SAM), the universal methyl donor; combining methionine with adenosine triphosphate (ATP) results in the “activated” SAM molecule. SAM is responsible for reactions that require the transfer of methyl groups to oxygen or nitrogen atoms of the acceptor molecule.^[^ ^32^ ^]^ When SAM donates a methyl group, it consequently forms S‐adenosylhomocysteine (SAH), which can then be further hydrolyzed to form homocysteine and adenosine.^[^ ^29^ ^]^ SAM is critical for the maintenance of methylation patterns in DNA.^[^ ^33a,b^ ^]^

**Figure 3:**
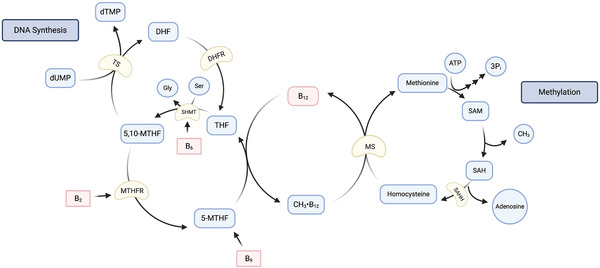
The one‐carbon metabolism pathway. Serine donates a methyl group to THF to create 5,10‐MTHF, leaving glycine. This (reversible) reaction is catalyzed by serine hydroxymethyltransferase (SHMT), using vitamin B_6_ as a cofactor. 5,10‐MTHF is converted to 5‐MTHF by methylenetetrahydrofolate reductase (MTHFR), with vitamin B_2_ as a cofactor and NADPH as electron donor (not depicted). 5‐MTHF, which can also be acquired from dietary folate (B_9_), donates a methyl group to B_12_ to create CH_3_•B_12_, a cofactor for the enzyme methionine synthase (MS), which catalyzes the conversion of homocysteine to methionine, by in turn transferring this CH_3_ group. Methionine is combined with ATP to become SAM, which maintains methylation reactions. When SAM donates a methyl group, it becomes SAH, which can be further hydrolyzed to form homocysteine and adenosine (by S‐adenosylhomocysteine hydrolase; SAHH). THF, left after CH_3_•B_12_ creation, can be converted back into 5,10‐MTHF. 5,10‐MTHF is a carbon donor for the enzyme thymidylate synthase (TS), which catalyzes the conversion of deoxyuridine monophosphate (dUMP) to deoxythymidine monophosphate (dTMP); hence, playing a role in de novo DNA synthesis. Upon this reaction, 5,10‐MTFH is converted into dihydrofolate (DHF), which can be reduced by dihydrofolate reductase (DHFR), using NADPH, regenerating THF. B‐family vitamins coming from food indicated in red. Based on Friso et al., 2017 and Green et al., 2017.^[^ ^13^, ^15^ ^]^

B_12_ works in unison with vitamin B_9_ (folate), vitamin B_6_, and vitamin B_2_ in the one‐carbon metabolism pathway. ^[^ ^13^, ^34^ ^]^ All four B vitamins act as cofactors for enzymes which participate in the pathway. The coenzyme form of folate, tetrahydrofolate (THF), acts as the primary acceptor for one‐carbon groups. A methyl group from serine is transferred to THF in order to form 5,10‐methylenetetrahydrofolate (5,10‐MTHF). This process is catalyzed by serine hydroxymethyltransferase with vitamin B_6_ as a cofactor. 5,10‐MTHF can then be further utilized in the de novo formation of thymidine, the synthesis of purines, as well as in the re‐synthesis of methionine.^[^ ^13^ ^]^ B_12_, in the form of CH_3_•B_12_, is a cofactor for the enzyme MS, the enzyme responsible for catalyzing the central reaction of the one‐carbon pathway. The steps are as follows: (1) a methyl group from 5‐methyltetrahydrofolate (5‐MTHF), the predominant form of dietary folate, is transferred to B_12_ to form CH_3_•B_12_; (2) this methyl group is then transferred to homocysteine by MS, resulting in the formation of methionine; (3) the remaining tetrahydrofolate (THF) can then be converted to 5,10‐MTHF, which can be further reduced by methylenetetrahydrofolate reductase (MTHFR), a vitamin B_2_ containing enzyme, to become 5‐MTHF (**Figure** **3**).^[^ ^13^, ^15^, ^26^, ^28^ ^]^

## 3 B_12_ Deficiency

Due to the essential role the vitamin plays, insufficient levels of B_12_ can be highly detrimental to one's health.^[^ ^19^ ^]^ The European Food Safety Authority (EFSA) Panel on Dietetic Products, Nutrition and Allergies concluded that the average adult requires a daily intake of 4 µg, and a pregnant woman requires up to 6 µg of B_12_.^[^ ^35^ ^]^ Despite this, individual intake typically consists of 2.4 µg day^‐1^ of B_12_, from which approximately 50–60% of the vitamin is absorbed.^[^ ^32^, ^36^ ^]^ Typically, a healthy individual has a serum level of approximately 200–900 pg mL^‐1^ (147‐662 pmol L^‐1^) of B_12_. A patient is considered deficient when they have less than 200 pg mL^‐1^ of serum B_12_, and critically deficient when they have 150 pg mL^‐1^ (111 pmol L^‐1^) or less.^[^ ^37^ ^]^ However, it should be noted that a B_12_ deficiency may be difficult to diagnose and the lower limit of B_12_ serum level may vary.^[^ ^38^ ^]^ Also, liver damage, e.g., stemming from alcoholism, as well as systemic low‐grade inflammation, may temporarily allow B_12_ serum levels to rise due to loss from liver stores, masking real deficiency.^[^ ^39^ ^]^

There are multiple ways to try to assess B_12_ status. Largely, it is determined through an analysis of total serum B_12_. However, this method often lacks specificity in the early stages of the deficiency. Due to this, other tests scoring B_12_ analytes have been developed in order to assess B_12_ status. When B_12_ binds to TC it is called holo‐TC.^[^ ^40^ ^]^ Some studies observed that the serum holo‐TC concentrations sufficiently reflect B_12_ status with an increased degree of specificity compared to measuring serum B_12_ levels. Of note, in plasma, B_12_ is not only found bound to TC, but to HC as well. In addition, multiple studies that examine the effects of a B_12_ deficiency also measure homocysteine and MMA concentrations (which will increase because the enzymes converting these compounds need B_12_ as a co‐factor), to achieve higher accuracy of results, as well.^[^ ^39^ ^;^ ^41^, ^42^, ^43^ ^]^ In order to strenuously evaluate and assess the possibilities of a B_12_ deficiency, researchers may thus choose to conduct multiple analyte testing and test for two or more measurements of B_12_ status simultaneously. An improved indicator of B_12_ status analysis seems to have been proposed by Fedosov et al. (2015), encompassing combined measurements of serum B_12_, plasma holo‐TC, homocysteine, and MMA. ^[^ ^44^ ^]^ Altogether, an accurate diagnosis of this deficiency is necessary to further reveal the presence or absence of correlations between potential clinical manifestations and respective target groups.

## 4 Vitamin B_12_ and Carcinogenesis

Carcinomas are cancers derived from epithelial tissue, which make up approximately 80% of all human cancer cases.^[^ ^45^ ^]^ Past studies have postulated that there might be a causal relationship between a localized insufficiency of B_12_ and carcinomas.^[^ ^46^ ^]^ In support of this hypothesis, Wu et al. (1999) performed a nested case‐control study and saw that in both menopausal and post‐menopausal breast cancer patients, there were significantly lower concentrations of serum B_12_, and patients who possessed the lowest B_12_ had an increased risk of breast cancer.^[^ ^47^ ^]^ This inverse association of serum levels of B_12_ and the risk of cancer is also evident in patients with cervical cancer. Pathak et al. (2014) found that cervical cancer patients had significantly lower serum B_12_ concentrations when compared to control patients.^[^ ^48^ ^]^ Furthermore, there was a significantly higher risk of human papillomavirus (HPV) infection when B_12_ levels were insufficient. HPV infection has been implicated in the etiology of 70% of cervical cancers. ^[^ ^49^ ^]^

This inverse relationship between B_12_ serum concentration and cancer risk is further seen in cancers of the gastrointestinal tract,^[^ ^50^, ^51^ ^]^ the liver,^[^ ^33a,b^ ^]^ the colon, and the rectum.^[^ ^52^, ^53^ ^]^ An overview of studies into relationships between B_12_ serum concentrations and cancer risk is given in **Table** **1**.

**Table 1:** Studies highlighting a relationship between B_12_ and cancer risk

Cancer type	Author (Year)	Outcome	Serum B_12_ concentration [pg mL^‐1^]
Breast	Wu et al. (1999) ^[^ ^47^ ^]^	Significantly higher risk of breast cancer	<280
Cervical	Pathak et al. (2014) ^[^ ^48^ ^]^	Significant inverse correlation between homocysteine concentration and B_12_ Lower serum B_12_ in patients with CIN or cervical cancer Higher risk of HPV infection	(a) 376.54^a)^ (b) 341.36
Gastric	Murphy et al. (2016) ^[^ ^50^ ^]^	Significantly increased risk of non‐cardia gastric adenocarcinoma and gastric carcinoid tumors	<200^b)^
	Miranti et al. (2017)^[^ ^51^ ^]^	5.8‐fold times significantly increased risk of developing non‐cardia gastric adenocarcinoma B_12_ can serve as a biomarker for atrophic gastritis that precedes non‐cardia gastric adenocarcinoma	<291–300
Hepatic	Brunaud et al. (2003)^c)^ ^[^ ^33a,b^ ^]^	Reduced MS activity and DNA methylation Increased risk of hepatocellular carcinoma	<200
Colorectal	Dahlin et al. (2008)^[^ ^52^ ^]^	Inverse association between plasma B_12_ levels and risk of rectal cancer	≤221
	Sun et al. (2015) ^[^ ^53^ ^]^	Insignificant reduction in colorectal cancer risk when B_12_ intake was below 12.8 µg d^‐1^ A slight reduction of colorectal cancer risk with every 4.5 µg d^‐1^ increment vitamin intake	‐ ^d)^

^a)^ Median serum B_12_ concentrations of (a) patients who were diagnosed with CIN while (b) correlates with patients diagnosed with cervical cancer;

^b)^ All subjects were patients with pernicious anemia;

^c)^ Animal study with F344 rats;

^d)^ Average serum B_12_ concentration not indicated

Certain studies also suggest onco‐protective properties of B_12_. Zhang et al. (2003) recorded that higher levels of B_12_ (>572.7 pg mL^‐1^) were significantly associated with a lower risk of breast cancer amongst premenopausal women.^[^ ^39^ ^]^ Similar results were obtained in a cervical cancer study. Piyathilake et al. (2009) reported that sufficient concentrations of B_12_ (≥200.6 pg mL^‐1^) correlated with a 50% reduction in cervical intraepithelial neoplasia (CIN) grade 2 diagnosis.^[^ ^54^ ^]^ Studies have implicated CIN in the pathogenesis of cervical cancer. In essence, low‐grade lesions (i.e., CIN1) are primarily associated with benign tumors and higher‐grade lesions (i.e., CIN2, CIN3) are associated with highly malignant cervical cancer cases.^[^ ^55^ ^]^ Interestingly, women who had low plasma folate concentrations but sufficient concentrations of B_12_ remained 50% less likely to be diagnosed as CIN2 positive.^[^ ^54^ ^]^ This inverse relationship is also apparent with regards to colorectal cancer risk and was further highlighted in a dose‐response meta‐analysis done by Sun et al. (2015). These authors observed a significant relationship between the dietary intake of B_12_ and colorectal cancer risk. They found a slight reduction in colorectal cancer risk with every 4.5 µg increase in dietary B_12_ (µg d^‐1^) intake.^[^ ^53^ ^]^ Research by Banjari and Hjartaker (2018) further confirmed this observation.^[^ ^56^ ^]^ Last, we should point out that MMA, which increases upon B_12_ deficiency, seems generally upregulated in serum of the elderly and, as was very recently found, might mediate tumor progression via its induction of SOX4 expression.^[^ ^57^ ^]^

## 5 Possible Mechanisms

Two possible mechanisms could especially explain the role of B_12_ in the etiology of some cancers. As previously mentioned, B_12_ plays a role in the central pathway of the one‐carbon metabolism cycle, which results in the formation of crucial components for the maintenance of DNA synthesis, repair, and methylation. An insufficient amount of B_12_ could lead to a disruption in this pathway, hence resulting in potentially adverse effects.^[^ ^32^ ^]^ When B_12_ levels are inadequate, MS activity is downregulated, hence impairing the remethylation of homocysteine to methionine. As a result, there is a decrease in methionine and subsequently SAM, as well as a concomitant increase in homocysteine. Such a downregulation of SAM could have detrimental effects for both DNA synthesis and repair, as well as for DNA methylation, possibly explaining some of the findings summarized in Tables 1 and 2.

### 5.1 B_12_ and DNA Synthesis

**Figure 4:**
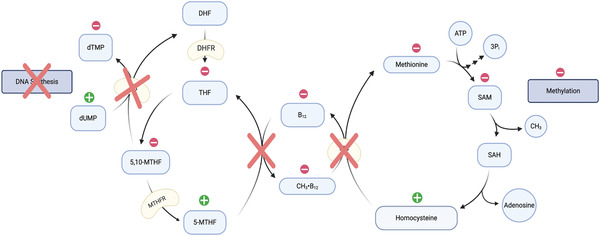
The methyl‐trap hypothesis. B_12_ deficiency impairs MS activity. MS is downregulated and homocysteine cannot be converted to methionine, hence resulting in an increase of homocysteine. This leads to a decrease in SAM production and global hypomethylation. Reduced SAM levels also manifest as a deficiency in THF. This results in a decrease of 5,10‐MTHF, the carbon donor for TS and an accumulation of dUMP, which may lead to increased misincorporation of uracil into DNA strands and halted DNA synthesis. Compare with Figure 3. Green “+” indicates rises in steady state concentrations; Red “–” in general indicates lower steady state concentrations; Based on Choi, 1999 and Lieberman and Marks, 2013.^[^ ^30^, ^58^ ^]^

A deficiency in B_12_ disrupts deoxythymidine monophosphate (dTMP) biosynthesis, necessary for DNA synthesis, due to the downregulation of essential precursors for dTMP synthesis. Reduced SAM levels, as a result of deficient B_12_, manifest as a deficiency in THF as the methyl group from 5‐MTHF becomes “trapped”; this model is called the methyl‐trap hypothesis.^[^ ^30^, ^58^ ^]^ Consequently, this results in a decrease of 5,10‐MTHF, the precursor to 5‐MTHF and the carbon donor for thymidylate synthase (TS); see **Figure** **4**. TS methylates deoxyuridine monophosphate (dUMP) to become dTMP, which is further phosphorylated to become deoxythymidine triphosphate (dTTP), an essential precursor for DNA synthesis and repair_._^[^ ^59^, ^60^ ^]^ Thus, when the enzyme cannot function properly, it leads to a downregulation of deoxythymidine production and an imbalance in the dUTP/dTTP ratio, which could culminate in an increase of uracil misincorporation in DNA (again, see Figure 4).^[^ ^61^ ^]^ When uracil is misincorporated, it results in the recruitment of the enzyme uracil‐DNA glycosylase that can recognize and excise the incorrect base by creating single‐strand or double‐strand breaks (DSBs) in the DNA.^[^ ^62^ ^]^ Repair pathways for DSBs are difficult and error‐prone; thus, leading to further complications such as genomic instability, increased mutagenesis, chromosomal breakages, and eventually apoptosis.^[^ ^63^ ^]^ All of these enhance an individual's susceptibility to developing cancer.^[^ ^50^ ^]^ Hence, deficient B_12_ levels could lead to a domino effect, culminating in an increased risk of developing cancer.

The presence of chromosomal damages was first seen in the form of “Howell‐Jolly bodies” in erythrocytes of patients with megaloblastic anemia, a clinical manifestation of a B_12_ deficiency.^[^ ^19^ ^]^ Other studies (both in vivo and in vitro) have also highlighted the association of low serum B_12_ with an increase of chromosome damage and micronucleus formation.^[^ ^41^, ^64^ ^]^ An animal study conducted by Choi et al. (2004) illustrated a causal relationship between a B_12_ deficiency, anomalies in base substitution, and colorectal carcinogenesis. The study showed that in the colonic mucosa of rats, after 10 weeks of consuming a diet low in B_12_, there was a 105% increase of uracil incorporation in colonic DNA. In order to establish an independent association, dietary folate and total folate was measured throughout the experiment. No depletion of folate in the colon was evident; thus, supporting the assumption that the base‐insertion anomalies occurred independently of folate levels.^[^ ^65^ ^]^

### 5.2 Vitamin B_12_ and DNA Methylation

In addition to effects in DNA synthesis, insufficiency of B_12_ also leads to aberrant DNA methylation. DNA cytosine methylation is an epigenetic mechanism which involves the transfer of a methyl group to the 5‐carbon position of CpG dinucleotides by DNA methyltransferases (DNMTs), the activity of which is stimulated by SAM. CpG dinucleotides refer to parts of the genome in which a cytosine nucleotide is immediately followed by a guanine nucleotide in the 5’ to 3’ direction.^[^ ^66^ ^]^ Chemical exposure and nutritional status have both been implicated in the alteration of methylation patterns.^[^ ^67^ ^]^ Broadly speaking, when DNA is hypermethylated, chromatin is compacted and consequently, locally encoded genes are no longer expressed. Conversely, when DNA is hypomethylated, chromatin unravels, and genes can become expressed.^[^ ^13^ ^]^ This is particularly dangerous as it could result in the activation of transposable elements,^[^ ^23^ ^]^ which are highly mutagenic and have been linked with multiple instances of cancer.^[^ ^68^ ^]^ Hence, dysregulation of this process can result in changes in gene expression independent of the primary DNA sequence.^[^ ^13^ ^]^ When B_12_ is lacking, MS is unable to catalyze the conversion of homocysteine to methionine, leading to an upregulation of homocysteine, and subsequently reduced synthesis of SAM (Figure 4). This limits available methyl groups and results in global hypomethylation of DNA, which may result in changes in gene expression. ^[^ ^13^, ^33^ ^]^ Global hypomethylation has been proven to be a risk factor for a variety of cancer types and is commonly seen during early carcinogenesis. ^[^ ^69^, ^70^, ^71^, ^72^ ^]^

The causative association of B_12_, global hypomethylation, and carcinogenesis has been studied extensively with regards to cervical cancer. As stated previously, HPV infection has been heavily implicated in the etiology of cervical cancer. However, it is important to note that only about 10% of all HPV infection cases lead to the development of CIN and only 8% of these cases eventually lead to cervical cancer. The transformative ability of HPV relies heavily on the expression of two viral oncoproteins, E6 and E7. In vitro studies have shown that cervical cancer cells undergo apoptosis when E6 and E7 are not expressed.^[^ ^73^ ^]^ According to Huang et al. (2018), B_12_ and folate work synergistically to maintain the methylation of the HPV E6 promoter and enhancer; thus, blocking the integration of HPV into the host genome.^[^ ^10^ ^]^ When B_12_ is deficient, a decrease in methylation of HPV‐16 E6 promoter and enhancer sites occurs, as reported by Piyathilake et al. (2014).^[^ ^74^ ^]^ Of note, HPV‐16 is one of the most frequent causative strains for cervical and anal cancer.^[^ ^75^ ^]^ It was observed that patients with less than 406.58 pg mL^‐1^ of B_12_ displayed a substantial decrease in HPV‐16 E6 methylation and were 37% more likely to develop CIN3. Inversely, a high concentration of serum B_12_ led to an increase of methylation of the E6 promoter and a 60% reduction of being diagnosed with CIN2. Again, a weak correlation between serum B_12_ and serum folate in this experiment also seems to establish that the two nutrients work independently in modulating the methylation of HPV and increasing the risk of CIN.^[^ ^73^ ^]^ Similarly, Ragsudha et al. (2011) found that having deficient B_12_ and folate levels increased the risk of developing CIN1 (about 15‐fold) and cervical cancer (approximately 9‐fold).^[^ ^76^ ^]^

Comparable trends of aberrant methylation have been identified in cases of hepatic, ^[^ ^33a,b^ ^]^ lung, ^[^ ^77^, ^78^ ^]^ colorectal, ^[^ ^23^, ^65^ ^]^ and head and neck cancer (**Table** **2**).^[^ ^79^ ^]^ Here, it should be pointed out that in this last example the correlation between B_12_ levels, methylation and cancer risk is not straight forward. In this instance, high B_12_ intake correlated with lower amounts of local (inactivating) tumor suppressor gene methylation, and thus lower cancer risk.

**Table 2:** Studies highlighting correlations between B_12_ deficiency and aberrant DNA methylation

Cancer type	Author (Year)	Outcome
Cervical	Piyathilake et al. (2014)^[^ ^74^ ^]^	Patients with less than 406.58 pg mL^‐1^ of B_12_ had less HPV‐16 E 6 promoter methylation 37% chance more likely to develop CIN3 A high concentration of serum B_12_ led to increased methylation of E6 promoter and 60% reduction in risk of being diagnosed with CIN2 Weak correlation between serum B_12_ and folate
	Ragasudha et al. (2012)^[^ ^76^ ^]^	<160 pm mL^‐1^ B_12_ serum concentration led to 14.9 times increased risk of developing CIN1 <160 pm mL^‐1^ B_12_ serum concentration led to 8.72 times increased risk of developing cervical cancer Dependent on low folate levels
Hepatic	Brunaud et al. (2003) ^[^ ^33^ ^a‐b]^	Low B_12_ activity led to reduced methionine synthase activity Low methionine synthase activity and low B_12_ led to DNA hypomethylation Livers of rats fed a B_12_ deficient diet showed methylation patterns similar to animals exposed to chemical carcinogens
Lung	Piyathilake et al. (2000)^[^ ^77^ ^]^	Localized deficiency of folate and B_12_ made cells more susceptible to carcinogens present in tobacco smoke Smokers have lower serum B_12_ levels than non‐smokers B_12_ levels are lower in squamous cancer cells (3.98 ± 1.3 pg µg^‐1^ DNA) than in adjacent non‐infected tissue (8.83 ± 1.3 pg µg^‐1^) Lower DNA methylation in squamous cell cancer cells Decreased activity of MTHR
	Johanning et al. (2002)^[^ ^78^ ^]^	Lung squamous cell cancer tissues had localized deficiencies of both folate and B_12_ These cells also had global DNA hypomethylation
Colorectal	Choi et al. (2004)^[^ ^65^ ^]^	Impaired DNA methylation was present in colonic tissue of colorectal cancer cells in rats Supplementation of B_12_ alleviated the deficiency after 10 weeks
	Hasan et al. (2019)^[^ ^23^ ^]^	B_12_ led to increased homocysteine levels Increased homocysteine levels led to an increase of cellular proliferation in Caco‐2 cell lines^a)^
Head and Neck	Colacino et al. (2012)^[^ ^79^ ^]^	Individuals with high B_12_ intake (32 µg d^‐1^) showed the least amount of tumor suppressor gene methylation

^a)^ A cell line of human epithelial colorectal adenocarcinoma cells.

## 6 Mitigating and Preventative Treatments

The evidence mentioned, illustrates a possible link between cancer and B_12_ deficiencies. Thus, we want to highlight possible preventative and mitigating treatments for the deficiency. As an individual's capability of absorbing B_12_ deteriorates with age, it might be vital to begin preventative measures early.^[^ ^80^ ^]^ Here, it must be said that to date, there are no well controlled studies showing that B_12_ supplements, as such, lower the risk of cancer in general and studies of different cancer types give conflicting results. ^[^ ^81^, ^82^ ^]^ However, extensive comparative studies regarding oncogenesis specifically in vegans are not available either.

To successfully apply early intervention, a diagnostic system for a B_12_ deficiency is important. Presently, the available on‐demand tests to diagnose the deficiency are serum B_12_, homocysteine, and MMA blood tests (see above). However, these diagnostic measures are typically only recommended for elderly patients or patients who demonstrate symptoms consistent with a deficiency.^[^ ^12^ ^]^ In light of the shifted trend of diet, incorporation of such a diagnostic test in the routine blood analysis for teenagers and young adults, in case they follow a restrictive diet, would in principle allow for effective mitigation and potentially prevent clinical impacts of B_12_ deficiencies at later (st)ages.

As for preventative measures, there are currently two techniques available to avert a B_12_ deficiency: supplementation and fortification. With regards to B_12_ supplementation, there are four routes of administration utilized: oral, nasal, sublingual, and intramuscular.^[^ ^28^ ^]^ The United States predominantly utilizes B_12_ in the form of cyanocobalamin, while hydroxocobalamin is predominantly used in Europe. ^[^ ^83^ ^]^ Originally, the intramuscular delivery was the preferred method of supplementation for a B_12_ deficiency, and remains such for clinical B_12_ deficiencies caused by malabsorption. However, due to the invasive nature of an intramuscular injection, and its dependency on the patient having access to a medical facility, as well as higher costs, this method can lead to low compliance.^[^ ^83^, ^84^ ^]^

Currently, oral B_12_ supplements are the preferred route of B_12_ supplementation for B_12_ deficiencies that result from nutritional deficiencies or low consumption. Some studies have found that B_12_ supplementation via oral administration (whether cyanocobalamin or hydroxocobalamin) had similar efficacies as intramuscular injections.^[^ ^85^, ^86^ ^]^ However, as these studies were limited in number and quality, doubts regarding the replacement of intramuscular injections linger. A widely cited panel by The Institute of Medicine (US) Standing Committee on the Scientific Evaluation of Dietary Reference Intakes (1998) suggests that a small dosage of 5 µg per day of oral cyanocobalamin at a time would be sufficient in ameliorating the deficiency.^[^ ^87^ ^]^ Others have suggested that rather a daily oral dosage of 10 µg of B_12_ is sufficient.^[^ ^4^ ^]^ Despite the difference in numbers, supplementation is generally advised for individuals who consume a diet low in B_12_ and may have a positive impact on public health ^[^ ^88^ ^]^ and possibly reduce the risk of developing cancer (but see above).^[^ ^61^ ^]^

Although not commonly used, B_12_ supplements can be administered nasally and sublingually as well. Results from these two administration methods have been positive in ameliorating a B_12_ deficiency. ^[^ ^89^, ^90^, ^91^ ^]^ Despite initial success, more research needs to be done to further test the efficacy and effectiveness of these methods in comparison with that of oral B_12_ supplements and B_12_ injections. Furthermore, vegans may choose to consume non‐animal B_12_ supplements, such as those derived from algae. However, supplements made from algae (e.g., spirulina) have been under debate, and several studies have found that many supplements produced from cyanobacteria can be labeled as “pseudovitamins”, without bioavailability for humans. ^[^ ^34^, ^92^ ^]^ Whether such supplements might further complicate clinical presentations by a residual influence on serum detection and/or unpredictable effects on B_12_ dependent pathways remains to be seen.

Another method of ensuring adequate intake of B_12_ for individuals partaking in a vegan diet would be consuming B_12_ fortified food products. Typically, fortification of food is preferred over oral supplementation due to its cost‐effectiveness.^[^ ^93^ ^]^ Currently, no country has implemented a fortification plan for B_12_,^[^ ^94^ ^]^ but products such as nutritional yeast, B_12_ fortified plant‐based milk, and a variety of meat analogues have been marketed to successfully alleviate a possible B_12_ deficiency.^[^ ^95^ ^]^ In light of the lack of any structural implementation so far, concomitant with the rising percentage of individuals partaking in a vegan diet, concerns have been raised regarding the need for a fortification plan.^[^ ^96^ ^]^ One specific problem associated with general fortification of food must be mentioned: for those with specific B_12_ resorption issues (see above) normal serum B_12_ levels might mask cellular deficiencies. This does not invalidate the need for fortification, but physicians should be much more acutely aware of this. When, even though serum B_12_ levels react positively to diet changes, typical neurocognitive symptoms indicating a deficiency persist, B_12_ might still be the problem. Future development of a robust B_12_ dependent enzymatic assay with cell extracts might put a stop to such uncertainties.

Different research groups have proposed different vehicles for B_12_ fortification to see which option would be the most feasible. Winkels et al. (2008) suggested that the implementation of flour co‐fortified with 9.6 µg B_12_ and 138 µg folic acid would be beneficial in raising serum B_12_ concentrations and providing necessary dietary requirements. They found that after 12 weeks, the consumption of fortified bread successfully improved serum B_12_ levels by 49%, decreased homocysteine concentrations by 13%, and MMA concentrations by 10%.^[^ ^97^ ^]^ Comparably, Siebert et al. (2017) looked into fortifying toothpaste with B_12_ for vegans in order to ensure adequate intake of the vitamin and found similar results. In addition to high compliance within groups of participants, the toothpaste was successful in raising both B_12_ serum concentrations, while simultaneously lowering homocysteine and MMA concentrations.^[^ ^91^ ^]^ Further efforts have also been made in fortifying tempeh; a traditional, widely consumed, Indonesian food product made from fermented soybeans.^[^ ^98^ ^]^

## 7 Limiting Factors and Suggestions for Future Research

It is worth noting that although a B_12_ deficient diet seems to have carcinogenic effects, there exists contradictory research regarding its onco‐protective nature. Hence, the overconsumption of B_12_ is not recommended. ^[^ ^99^, ^100^, ^101^ ^]^ Some studies have suggested that once a threshold value is reached, a diet high in B_12_ could potentially lead to carcinogenesis as well. Patients who had greater than 1000 pmol of serum B_12_ (∼50% above the currently used upper “healthy” limit) had a higher risk of developing solid cancer.^[^ ^102^, ^103^ ^]^ A potential explanation for these findings could be that higher B_12_ levels led to the hypermethylation, and subsequently, the silencing of certain key genes (i.e., tumor suppressor genes; but see above). Local hypermethylation of promoter regions in tumor‐suppressor genes, genes involved in cell adhesion or apoptosis, and genes which encode for upstream regulators of essential processes, has been implicated to play a critical role in carcinogenesis.^[^ ^104^ ^]^ Hence, in the future, a follow‐up study should be conducted to see whether high B_12_ consumption past a certain threshold could prove to be cytotoxic and/or carcinogenic. Therefore, a meta‐analysis of the available global data or a large‐scale study to reach a consensus of the threshold level would be useful. Furthermore, relevant underlying molecular processes should be investigated. Here, a first, important but difficult question is of course whether such high levels of B_12_ are indeed carcinogenic/toxic by themselves, or can, e.g., be understood as markers for excessive food intake (with or without toxic contaminants).

In light of the potential implementation of diagnostic approaches to detect B_12_ deficiencies, currently the techniques generally used are heavily based on serum B_12_ levels. However, this is problematic as it was found to be unreliable in diagnosing certain clinical manifestations of a B_12_ deficiency.^[^ ^12^ ^]^ A further improvement of this diagnostic system would be beneficial in accurately mapping deficient patients and developing a potential mitigation strategy.

Another limitation is related to the greatly interconnected role B_12_ and folate play in the one‐carbon metabolism. Due to this, it is difficult to establish whether B_12_ works independently in potentially promoting or impeding carcinogenesis, or synergistically with folate. This is of particular interest with regards to a vegan diet, which is high in folate intake, while being deficient in B_12_.^[^ ^105^ ^]^ Additionally, studies have reported that high folate concentration could mask the symptoms of a B_12_ deficiency; thus, making it harder to diagnose quickly.^[^ ^106^ ^]^ Future studies should look into the effects of a diet high in folate and deficient in B_12_ and whether high folate intake could compensate for the lack of B_12_ and/or rather mask an existing deficiency.

Moreover, with regards to opportunities for future research, concurrent with the rise of further improved genome editing technology, a potential area of future research would be the application of these innovations to biofortify staple foods of the vegan diet with B_12_. This might prove to be a challenge as plants are not capable of synthesizing B_12_ on their own; thus, there would need to be a symbiosis between the staple crop of choice and B_12_ producing bacteria. ^[^ ^107^, ^108^ ^]^ Tweaking the relevant genes of the microbiome genome may improve B_12_ levels.

Comprehensive knowledge of the epigenome and its correlation with disease onset, developing a better diagnostic method for B_12_ deficiency, and establishing further mitigation and prevention strategies, while critically comparing B_12_ sources, may save some of the future generations of vegans from potentially developing cancer and other major diseases.

## 8 Conclusion

A growing body of research has shown that insufficient intake of B_12_ may have a role in carcinogenesis. Evidence indicates that a vegan diet can lead to a B_12_ deficiency and could possibly inadvertently lead to cancer. This is due to the critical role B_12_ plays in regulating crucial physiological processes, such as DNA synthesis and DNA methylation. A deficiency in B_12_ henceforth leads to base‐substitution anomalies and aberrant methylation patterns, leading to genomic instability and abnormal gene expression. Both have been implicated in the development of multiple forms of cancer. Due to the detrimental effects of a B_12_ deficiency, individuals who partake in a strict vegan diet need to ensure adequate intake through supplementation and/or the consumption of fortified products. However, in light of the existing evidence, the complex correlation between B_12_ and cancer needs further in‐depth studies. This is in part due to the multifactorial nature of cancer, the role of B_12_ in MMA conversion, and the interconnected nature of B_12_ and the other B vitamins.

## Conflict of Interest

The authors declare no conflict of interest.

## Data Availability

Data sharing is not applicable to this article as no new data were created or analyzed in this study.
